# Population pharmacokinetic study of pemetrexed in chinese primary advanced non-small cell lung carcinoma patients

**DOI:** 10.3389/fphar.2022.954242

**Published:** 2022-08-25

**Authors:** Peng Cao, Wei Guo, Jun Wang, Sanlan Wu, Yifei Huang, Yang Wang, Yani Liu, Yu Zhang

**Affiliations:** ^1^ Department of Pharmacy, Union Hospital, Tongji Medical College, Huazhong University of Science and Technology, Wuhan, China; ^2^ Hubei Province Clinical Research Center for Precision Medicine for Critical Illness, Wuhan, China; ^3^ Department of Clinical Pharmacy, Wuhan Children’s Hospital, Tongji Medical College, Huazhong University of Science and Technology, Wuhan, China

**Keywords:** pemetrexed, population pharmacokinetics, ERCC1, CYP3A5, polymorphisms, creatinine clearance, NSCLC

## Abstract

The purposes of this study were to identify physiological and genetic factors that contributed to variability of pemetrexed (PEM) exposure and to optimize the dosing regimens for Chinese non-small cell lung carcinoma patients. A prospective population pharmacokinetics (PPK) research was performed in this population. The PEM concentrations of 192 plasma samples from 116 in-hospital patients were detected. All patients were genotyped for polymorphisms. The PPK model of PEM was developed. The pharmacokinetic behavior of PEM was described by a two-compartment model with first-order elimination. The population typical values were as follows: clearance (CL) 8.29 L/h, intercompartmental clearance (Q) 0.10 L/h, central volume of distribution (V1) 18.94 L and peripheral volume of distribution (V2) 5.12 L. Creatinine clearance (CrCl) was identified as a covariate to CL, and ERCC1 (rs3212986) and CYP3A5 (rs776746) gene polymorphisms as covariates to Q. By using empirical body surface area (BSA)-based dosing strategy, PEM exposure decreased with the elevation of CrCl. Contrarily, CrCl-based dosing strategy exhibited a satisfactory efficacy of achieving the target PEM exposure. BSA-based dosing regimen in current clinic practice is not suitable to achieve the target exposure in PEM chemotherapy of Chinese NSCLC patients. Alternatively, renal function-based dosing strategy is suggested.

## Introduction

Lung carcinoma is the leading cause of cancer-related deaths globally with approximately 1.5 million annual deaths (Khan et al., 2021). Non-small cell lung carcinoma (NSCLC) accounts for 85% of all cases of lung carcinoma and the large majority of NSCLC patients are diagnosed at advanced stage with decreased 5-years survival rate. As an antifolate metabolic inhibitor, pemetrexed (PEM) is widely used in various tumors, especially NSCLC and mesothelioma (de Rouw et al., 2021b). Recently, immunotherapy in combination with PEM-based platinum or carboplatin showed a better survival benefit compared with chemotherapy alone (Gandhi et al., 2018), and this combined treatment has also been recommended as first-line treatment for advanced NSCLC (Ettinger et al., 2021). PEM is a multi-targeted drug, which acts mainly by suppressing thymidylate synthase and thus decreases the amount of thymidine available for DNA synthesis. In addition, PEM also suppresses the enzymes involved in purine synthesis and folate metabolism, including glycinamide ribonucleotide formyl transferase and dihydrofolate reductase.

With the wide application of PEM in clinic, the efficacy and safety of PEM have been paid increasing attention. In clinical practice, PEM is normally administrated by body surface area (BSA)-based dosing strategy, which leads to large inter-individual variability in PEM exposure (Visser et al., 2019). The corresponding reason is that the metabolic behavior of PEM *in vivo* is not directly related to BSA, and that there are individual differences in the *in vivo* activity of metabolic enzymes between patients (de Rouw et al., 2021a). It has been well acknowledged that PEM is not metabolized to an appreciable extent and is primarily eliminated by renal excretion as an unchanged form during the first 24 h after administration (de Rouw et al., 2021a). These findings indicate that PEM exposure in human body is closely related with individual renal function.

The conventional administration strategy based on BSA is likely to cause low blood drug concentration, thus affecting the efficacy of PEM. Besides, it may also result in high blood drug concentration, which leads to intolerable or even life-threatening adverse reactions in patients. In the single agent trials with PEM, the most common adverse reactions are neutropenia, thrombocytopenia, reversible elevation of transaminase level, anorexia, diarrhea, mucositis, nausea, rash, and asthenia (de Rouw et al., 2021b). Therefore, it is necessary to monitor the drug concentration and optimize the dose in PEM chemotherapy.

The population pharmacokinetics (PPK) analysis is a practical method to evaluate the pharmacokinetic characteristics and exposure of drug to obtain individual dosing in a target population (Buil-Bruna et al., 2016; Chen et al., 2017; Wang et al., 2020). PPK models of PEM in NSCLC patients in the Netherlands (Visser et al., 2019), India (Srinivasan et al., 2019) and the United States (Latz et al., 2006) have been reported and the clinical applicability of these models has been demonstrated. According to the comparison of typical PPK values of PEM and identified covariates in these studies, it is suggested that ethnic factor may play an important role in the behavior of PEM in human body of different populations. However, there are no PPK studies of PEM in Chinese primary advanced NSCLC patients. There are a large number of NSCLC patients in China, and PEM is often used for systemic treatment of patients with no driver gene or targeted therapy resistance. Therefore, there is an urgent need to discover the covariates that affect the disposition and metabolism of PEM in Chinese population and optimize the dosing strategy to avoid ineffective treatments.

The present study followed a sparse blood sampling strategy, along with the availability of rich data including a comprehensive single nucleotide polymorphism (SNP) gene locus. To the best of our knowledge, this is the first study to explore the potential gene polymorphisms that affect the physiological disposition process of PEM. Herein, we aimed to establish a PPK model of PEM to describe its pharmacokinetic behavior and to determine the physiological and genetic factors that lead to the variability of its exposure in Chinese patients with primary advanced NSCLC, which provided proof-of-concept for pharmacokinetically-guided dosing of PEM for them. Also, we proposed an easy-to-use renal function-based dosing strategy to help achieving the target PEM exposure of area under the concentration-time curve (AUC).

## Materials and methods

### Study participants

The PPK study of PEM was a prospective research, which was conducted at Wuhan Union Hospital from February 2018 to December 2019. A total of 116 patients were enrolled from the cancer center of Wuhan Union Hospital (Wuhan, Hubei, China). All the patients were histologically diagnosed with primary NSCLC and subjected to at least two cycles of PEM plus platinum (cisplatin/carboplatin/nedaplatin) based chemotherapy as primary treatment. The inclusion and exclusion criteria are described in the Supplementary Material. A total of 192 plasma samples (1–3 samples per patient) were collected at different times after PEM intravenous infusion. This study had been approved by the Ethics Committee of the Union Hospital, Tongji Medical College, Huazhong University of Science and Technology (2018-S332, date of approval: 2018.6.27). Written informed consent was obtained from all patients. All procedures followed the instructions of the Local Ethics Committee.

### Dosage regimen and pharmacokinetic sampling

The patients were dosed according to BSA and received a 15-min intravenous infusion of 500 mg/m2 of PEM dissolved in 100 ml saline at their first chemotherapy cycle. For each patient, one or two blood samples were randomly collected from the time points of 0.5, 1, 3, 5, 7, 24, 48 or 72 h after infusion. The heparin anticoagulated plasma samples were centrifugated at 3,500 rpm for 10 min at 4°C, followed by pipetting supernatant plasma and immediately transferring to a −80°C freezer until metabolic analysis.

The following data were collected through an electronic medical record system of hospital, including gender, age, BSA, albumin (ALB), alkaline phosphatase (ALP), alanine aminotransferase (ALT), aspartate aminotransferase (Kearns et al., 2003), gamma-glutamyl-transpeptidase (GGT), total bilirubin (TBIL), total bile acid (TBA), creatinine (Cre), blood urea nitrogen (BUN), uric acid (UA), white blood cell (WBC), neutrophils, hemoglobin (HGB), and platelet (PLT). Creatinine clearance (CrCl) was calculated by the Cockcroft-Gault equation and BSA was calculated according to the Mosteller equation as follows (Fancher et al., 2016):

$$BSA\left(m^{2}\right)=\sqrt{\frac{Ht\left(cm\right)\times Wt\left(kg\right)}{3600}}$$

### Analytical method of pemetrexed

The plasma concentrations of PEM were detected using an ultra-performance liquid chromatography-electrospray ionization tandem mass spectrometry (UPLC-ESI-MS/MS) system (UPLC, Shim-pack UFLC SHIMADZU CBM A system; MS, QTRAP^®^ System). The chromatographic separation was performed on an ACE C18 column (50.0 mm × 2.1 mm, 5 μm). The mobile phase consisted of water containing 0.2% formic acid (A) and acetonitrile containing 0.2% formic acid (B). The flow rate was 0.3 ml/min. The gradient program started from 10% B and increased linearly to 70% B in 1.20 min and further rose to 95% B from 1.20 to 1.60 min. After maintaining at 95% B for 0.60 min, it was brought back to 10% B within 0.10 min followed by 0.60 min re-equilibration. The injection volume was 5 μl for low calibration curve (2.5–600 ng⋅ml^−1^) and 20 μl for high calibration curve (0.6–120 μg⋅ml^−1^), respectively. The column and the autosampler temperature were maintained at 40°C and 4°C, respectively. The MS/MS detection was carried out in positive Electrospray Ionization (ESI) mode using MRM mode. The monitoring ion pair was 428.2→163.1 for PEM and 433.2→163.1 for PEM-d5 (isotope internal standard of PEM), respectively. This method had been strictly developed according to guidance document of Bioanalytical Method Validation Guidance for Industry (U.S. Food and Drug Administration). All items (including specificity, accuracy, precision, stability, etc.) met the testing standards (data shown in Supplementary Material).

### Microarray experiment and genotyping

DNA was extracted from blood samples of NSCLC patients by using QIAamp DNA Mini Kit (Qiagen). The DNA quality was evaluated by checking the OD260/OD280 ratio in spectrophotometer and integrity in agarose gels, with OD260/OD280 ratio between 1.8 and 2.0 and the DNA length greater than 10 Kb in size demonstrating an eligible quality. In the current study, 2 of the 118 initial samples were discarded due to quality control failure.

Genotyping was performed on Axiom2.0 platform by using Capital Biotechnology Precision Medicine Research Array (CBT-PMRA) Kit (Thermo Fisher Scientific, Waltham, MA, USA). The microarray contained over 787400 SNPs including 50,000 novel markers covering East and South Asian populations based on version 19 of the human genome (GRCH 37). The microarray experiments were performed according to the standard operating procedures (Axiom™ 2.0 assay 96-array format manual workflow user guide). The whole experiment consisted of five stages and lasted for four to 5 days. In brief, DNA amplification lasted for 24 h in the first stage, followed by DNA fragmentation and precipitation on the second day. Then, the DNA pellets were dried in the oven and dissolved by Resusp buffer. The DNA concentrations and fragment sizes were tested to assess whether they could be used in subsequent hybridization experiments. DNA hybridization, ligation, staining, washing and array scanning were all performed on the GeneTitan™ MC Instrument. AxiomTM analysis suite v4.0.1 was used to process genotyping.

### PPK modeling of pemetrexed

A non-linear mixed-effects model of PEM in Chinese primary advanced NSCLC patients was conducted by adopting the modeling program Phoenix® NLME (Version 8.2.0.4383, Pharsight Corporation, USA). The initial estimation of pharmacokinetic parameters was tested by referring to previous publication (Srinivasan et al., 2019) and adjusted by a naive-pooled method. Subsequent accurate pharmacokinetic parameters and their variability were estimated by using first order conditional estimation-extended least squares (FOCE-ELS) method. The PPK model consisted of a structural model and several random-effect models. The structural model was used to elucidate the relationship between concentration and time, and the random-effect models were used to assess the inter-individual and intra-individual variability of PPK.

### Base model development

One- or two-compartment models with linear or non-linear (Michaelis-Menten) elimination after the intravenous administration of PEM were investigated to determine the optimal structural model.

The intra-individual variability (also referred as residual variability) of PPK was initially described by using additive, proportional, combined and exponential models, respectively, where Y represented the observed PEM concentration, IPRED was the individual predicted concentration, and ε/ε′ was regarded to follow a normal distribution with a mean of 0 and a variance of σ^2^/σ′^2^. Different basic models were evaluated through the improvement of the objective function value (OFV, −2 * log-likelihood).

### Additive error model: Y=IPRED+ε

Proportional error model: Y=IPRED × (1+ε)

Combined error model: Y=IPRED × (1+ε) +ε′

Exponential error model: Y=IPRED × exp (ε)

The exponential model was used to describe inter-individual variability as follows, in which P*i* was the estimated parameter value of the individual *i*, θ represented the typical population parameter, and η*_i_* was assumed to be normally distributed with a mean of 0 and a variance of ω^2.

$$P_{i}=\theta \times \text{exp}\left(\eta_{i}\right)$$

### Covariate model development

Before covariate screening, we conducted correlation analysis between continuous covariates to avoid including collinearity variables in the model. A total of 17 continuous covariates (age, BSA, ALB, WBC, HGB, PLT, Neutrophils, ALP, ALT, AST, GGT, TBIL, TBA, BUN, Cre, UA, CrCl) were tested by using a spearman analysis in SPSS software (Supplementary Table S1). Significant correlation was indicated as asterisk. After removal of collinearity variables, age, BSA, ALB, WBC, ALP and CrCl were finally included in subsequent covariate screening process.

Categorical variables including gender, type of platinum agent (either cisplatin, carboplatin or nedaplatin) and a list of gene polymorphism SNP locus in the genes of SLC22A8, SLC22A9, SLC19A1, SLC22A11, ABCB1, ABCC2, CYP3A4, CYP3A5, CYP19A1, SLCO1B1, SLCO1B3, MTHFR, DHFR, TYMS, GGH, DCK, ATIC, FOLR3, XRCC1, RRM1, ERCC1, ERCC2 and ERCC5 were also included in the search of significantly correlated covariates (Supplementary Table S2).

The stepwise model was used to analyze the influence of each covariate on the model parameters, including forward inclusion and backward elimination procedures with likelihood ratio test. During forward inclusion process, a covariate was added if a significant decrease in the objective function value (OFV) was obtained (more than 3.84, *p* < 0.05). After all significant covariates were incorporated into a full model, backward elimination was applied to assess the importance of them. If the elimination of a covariate resulted in an increase of OFV that was less than 6.63 (*p* < 0.01), it would be excluded from the full model.

The effects of continuous covariates and categorical covariates were described as follows, respectively:

Continuous covariates: θi=θ×(Covj∕Covmean)^θCov

Categorical covariates: ${\text{θ}}_{\text{i}}=\text{θ}\times \exp\left({\text{θ}}_{\text{Cov}}\right)$ where ${\text{Cov}}_{\text{j}}$ represented the j-th covariate, ${\text{Cov}}_{\text{mean}}$ was the average value of the covariate, ${\text{θ}}_{\text{i}}$ represented the i-th population prediction of the parameter, θ was the population typical value of the parameter, and ${\text{θ}}_{\text{Cov}}$ was an estimated parameter describing the fixed effect of the covariate on the PPK parameter.

### Final PPK model validation

The performance of the final model was evaluated by goodness-of-fit (GOF) plots, non-parametric bootstrap and visual predictive check (VPC). GOF plots including observed concentrations (DV) vs. individual population prediction (IPRED)/population prediction (PRED) and conditional weighted residuals (CWRES) vs. PRED/time after dose were employed initially for diagnostic purposes. During the bootstrap process, resampling and replacement were repeated 1,000 times, and estimated parameters of bootstrap simulation were compared with their counterparts in the final model. In addition, the prediction performance of the final model was also assessed visually by VPC. Specifically, the final model was employed to simulate 1,000 predicted data sets, which were compared with observed concentrations. The 5th, 50th, and 95th percentiles of the predicted concentrations were calculated and plotted.

## Results

### Patients’ demographics and laboratory examination

**FIGURE 1:**
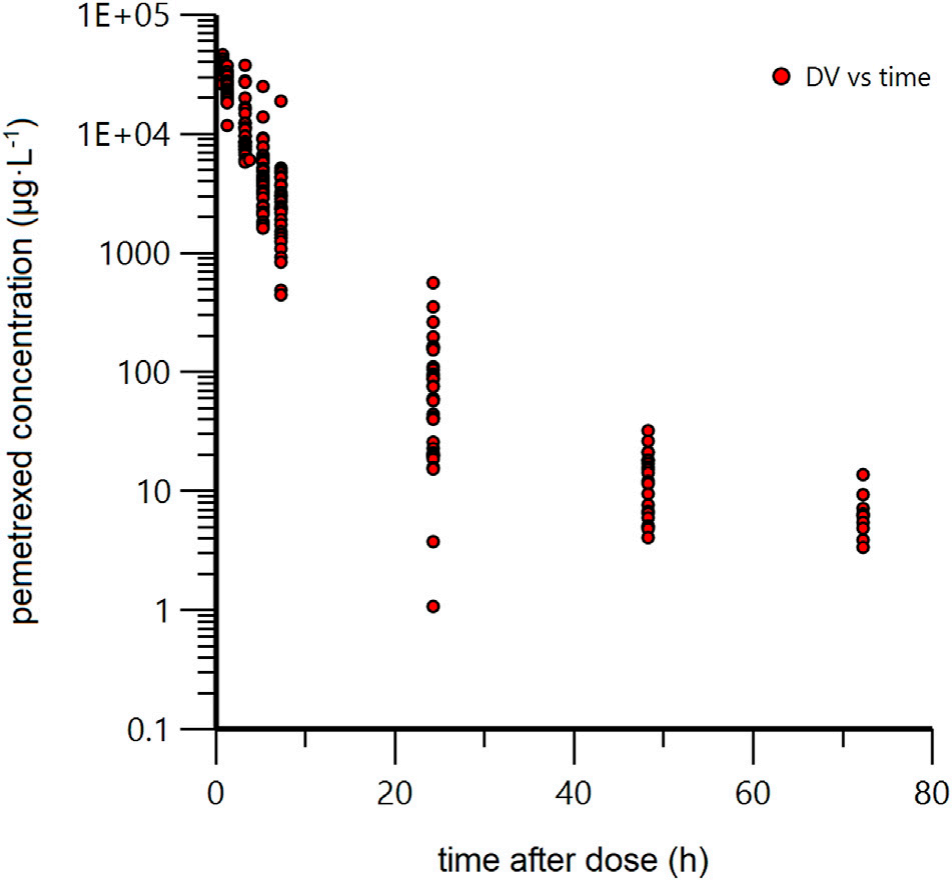
Scatterplot of concentration-time relationship for pemetrexed.

A total of 118 patients were initially enrolled in the study but two patients were excluded due to DNA degradation and lack of gene SNP results. Finally, 116 patients were included in the PPK analysis, and 192 PEM concentrations were obtained. The concentration-time profile of PEM was shown in Figure 1. The demographics and clinical features of the participants were summarized in Table 1.

**TABLE 1:** Clinical characteristics of 116 NSCLC patients.

	Mean value (SD)	Median value (range)
Patients (n)	116	
Samples (n)	192	
Gender (Male: Female)	69:47	
Platinum (cisplatin: carboplatin: nedaplatin)	43: 37: 31	
Age (years)	55.7 (9.6)	57.0 (27.0–73.0)
BSA (m^2^)	1.7 (0.1)	1.7 (1.4–2.0)
ALB (g/L, 35–55)	38.1 (4.4)	37.7 (27.4–50.9)
ALP (U/L, 40–150)	113.2 (92.6)	91.0 (31.0–730.0)
ALT (U/L, 5–40)	31.5 (31.5)	24.0 (8.0–195.0)
AST (U/L, 8–40)	27.7 (17.4)	23.0 (10.0–132.0)
GGT (U/L, 11–50)	42.6 (41.8)	29.0 (8.0–254.0)
TBIL (μmol/L, 5.1–19.0)	12.3 (4.4)	11.4 (5.4–26.3)
TBA (μmol/L, 0–10.0)	5.9 (12.0)	3.5 (0.2–117.8)
CRE (μmol/L, 44.0–133.0)	68.2 (16.4)	66.0 (36.8–123.5)
BUN (mmol/L, 3.2–7.1)	5.4 (1.7)	5.1 (2.6–11.4)
UA (μmol/L)	275.6 (92.5)	270.0 (24.0–584.0)
CrCl (ml/min)	93.6 (26.5)	89.5 (47.5–179.7)
WBC (g/L, 3.5–9.5)	8.9 (6.0)	7.0 (3.8–39.4)
Neutrophils (g/L, 1.8–6.3)	6.7 (6.1)	4.6 (1.7–37.0)
HGB (g/L, 130–175)	128.7 (14.3)	129.0 (94.0–163.0)
PLT (10^9/L, 125–350)	241.5 (69.0)	241.0 (91.0–453.0)

### PPK base model development

The PPK analysis was based on 192 PEM plasma concentrations from 116 primary advanced NSCLC patients. According to previous findings, a two-compartment structural model with constant rate input (intravenous infusion) and first-order elimination could adequately describe the concentration-time points of PEM (Latz et al., 2006; Srinivasan et al., 2019). The model was parameterized according to central volume of distribution (V1), peripheral volume of distribution (V2), clearance (CL) and intercompartmental clearance (Q) of PEM.

To illustrate the intra-individual variability of the PPK, four kinds of models were evaluated by comparing the OFV and finally a proportional model with the smallest OFV was employed. In addition, the inter-individual variability was described by using an exponential model, which had been mentioned earlier.

### PPK covariate model development

After establishing the basic model, we aimed to search for the covariates that affected the PPK of PEM. In this study, we collected a total of 17 continuous covariates and 45 categorical covariates. Before covariate screening, we performed a spearman correlation analysis on continuous covariates to ensure that collinearity covariates (*p* < 0.05) were not included in the development of the final PPK model simultaneously.

After screening potential covariates using a stepwise procedure, we found that CrCl was an important covariate for CL whereas CYP3A5 (rs776746) and ERCC1 (rs3212986) polymorphisms significantly affected Q. V1 and V2 were not affected by any of these covariates. Of note, since most NSCLC patients (111 of 116 patients) in this study were treated with PEM combined with a kind of platinum (cisplatin/carboplatin/nedaplatin), we included different platinum drugs as categorical variables into the analysis but our results showed that different kinds of platinum did not affect any of the parameters in this model.

**FIGURE 2:**
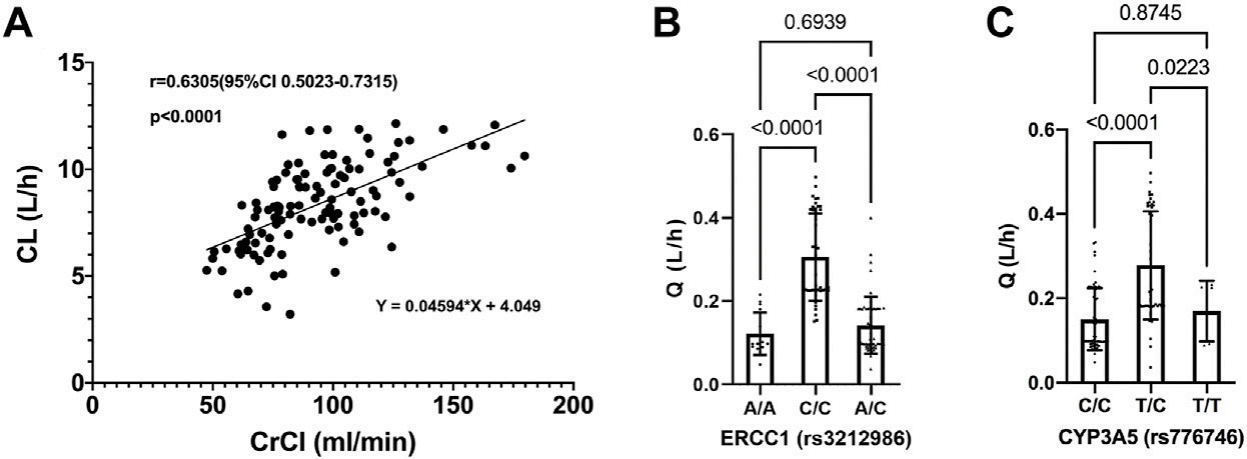
Relationship between pemetrexed clearance and covariates. **(A)** Pemetrexed clearance vs. CrCl, a spearman correlation analysis is used to fit the scatter plot; **(B)** Pemetrexed intercompartment clearance vs. ERCC1 (rs3212986); **(C)** Pemetrexed intercompartment clearance vs. CYP3A5 (rs776746); Statistical difference is analyzed by one-way ANOVA.

As shown in Figure 2A, the CL of PEM increased with the elevation of CrCl, with a spearman analysis showing a strong correlation of them (r = 0.6187, *p* < 0.0001). In addition, polymorphism of ERCC1 gene showed a significant effect on Q, where patients with a C/C genotype in rs3212986 locus exhibited higher Q values than those with other genotypes (Figure 2B). Similarly, patients with a T/C genotype in rs776746 locus manifested higher Q values than the others (Figure 2C).

The final model was shown as follows:

$$V_{1}\left(L\right)={\text{θ}}_{V_{1}}$$

$$V_{2}\left(L\right)={\text{θ}}_{V_{2}}$$

$$CL\left(L/h\right)={\text{θ}}_{CL}\times {\left(\frac{CrCl}{CrCl_{Mean}}\right)}^{{\text{θ}}_{1}}\times \exp\left({\text{η}}_{CL}\right)$$

$$Q\left(L/h\right)=\theta_{Q}\times \exp\left({\text{θ}}_{ERCC1}\right)\times \exp\left({\text{θ}}_{CYP3A5}\right)\times \exp\left({\text{η}}_{Q}\right)$$

Where V1, V2, CL and Q represented the individual pharmacokinetic parameters, η was the random effect for CL or Q in different patients. θ was the fixed effect in each parameter. CrCl was creatinine clearance rate. θ of each parameter was shown in Table 2. If ERCC1 phenotype (rs3212986) = C/C, ${\text{θ}}_{\text{ERCC}1}={\text{θ}}_{2}$ (0.83); If ERCC1 phenotype (rs3212986) = A/C or A/A, ${\text{θ}}_{\text{ERCC}1}=0$ . If CYP3A5 phenotype (rs776746) = T/C, ${\text{θ}}_{\text{CYP}3\text{A}5}={\text{θ}}_{3}$ (0.62); If CYP3A5 phenotype (rs776746) = C/C or T/T, ${\text{θ}}_{\text{CYP}3\text{A}5}=0$ . ${\text{CrCl}}_{\text{Mean}}$ was the average value of CrCl (93.6). It was noteworthy that the random effects were excluded from V1 and V2 of final models due to their high shrinkage values (99.51% and 99.97%).

**TABLE 2:** Population pharmacokinetic parameters of PEM and bootstrap results (n = 1,000).

Parameter	Final model			Bootstrap analysis		Bias (%)
	Estimate	RSE (%)	IIV(*ω*%)	Median	95% CI	
V_1_ (L)	18.94	5.94	NA	18.96	17.53–20.85	0.11
V_2_ (L)	5.12	18.85	NA	5.15	3.86–6.36	0.59
CL (L·h^−1^)	8.29	4.43	5.61	8.30	7.63–8.95	0.12
Q (L·h^−1^)	0.10	18.03	24.17	0.10	0.08–0.13	0
${\text{θ}}_{1}$	0.58	26.90	NA	0.58	0.46–0.72	0
${\text{θ}}_{2}$	0.83	29.66	NA	0.78	0.38–1.15	-6.02
${\text{θ}}_{3}$	0.62	36.45	NA	0.64	0.11–0.99	3.23
$\eta_{CL}$ -shrinkage (%)	NA	13.89	NA	NA	NA	NA
$\eta_{Q}$ -shrinkage (%)	NA	47.06	NA	NA	NA	NA
Residual variability						
σ (%)	24.72	14.11	NA	24.68	18.15–32.13	-0.16
ε-shrinkage (%)	39.99	NA	NA	NA	NA	NA

CL, indicated clearance; Q, intercompartment clearance; V1, central volume of distribution; V2, peripheral volume of distribution. IIV, inter-individual variability; RSE, relative standard error; NA, not applicable.

The population parameter estimates (including V1, V2, CL, Q, inter-individual variability and residual variability), fixed effects and random effects of the final model were presented in Table 2. The population typical values of V1, V2, CL and Q were 18.94, 5.12, 8.29 L/h, and 0.10 L/h, respectively. After incorporation of covariates in the final model, the inter-individual variability was 5.61% and 24.17% for CL and Q, respectively, and the residual variability was 24.72%.

### Model evaluation and validation

**FIGURE 3:**
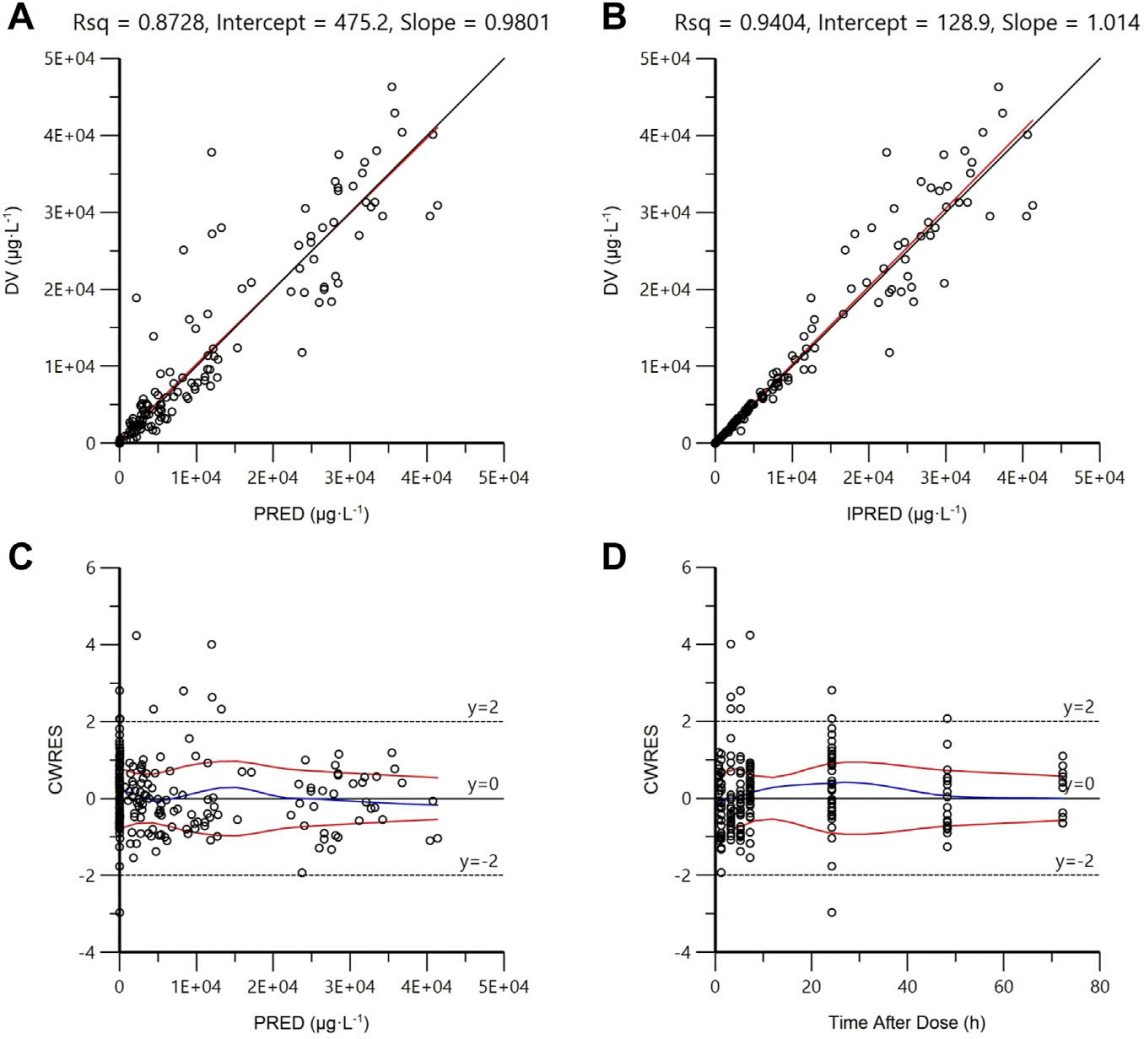
Goodness-of-fit plots of the final full model. **(A)** Observed concentrations (DV) vs. population prediction (PRED); **(B)** DV vs. individual prediction (IPRED); **(C)** Conditional weighted residuals (CWRES) vs. PRED; **(D)** CWRES vs. time after dose. The uniform line and the regression line are presented in **(A)** and **(C)**. The tendency curves of CWRES are shown in **(C)** and **(D)**.

As shown in Figures 3A,B, in diagnostic goodness-of-fit plots, both PRED and IPRED were close to the DV, which responded to an acceptable prediction accuracy of the final full model. Most CWRES equally laid within ±2 ranges (Figures 3C,D), demonstrating an accurate predicting ability of the final model.

**FIGURE 4:**
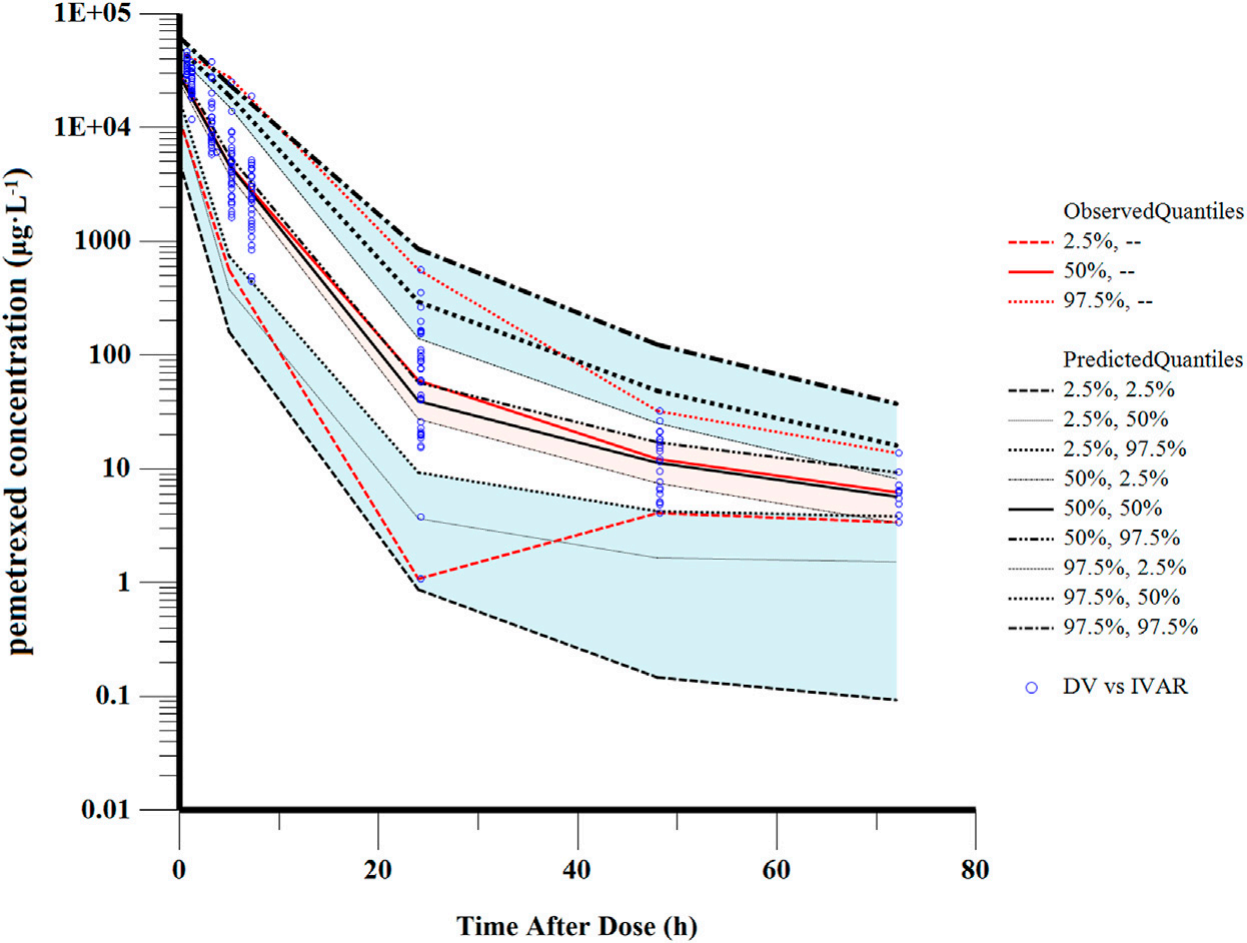
Visual predictive check of the final model. The solid red line represents the median observed concentrations. The solid black line represents the median simulated concentrations. The 2.5th and 97.5th percentiles of these concentrations are presented with dashed lines.

The good stability of the final model was confirmed by the results of 1,000 bootstrap analysis (Table 2). All parameters obtained from the final model were close to the respective median values of bootstrap estimation. The bias was defined as (median value of bootstrap model–estimate value of the final model)/(estimate value of the final model) × 100% and the bias values for all parameters were below ±10%, demonstrating the final full model was stable. The VPC examination was shown in Figure 4. Almost all observed concentrations were within the 95%CI regions, indicating a good predictive performance of the final model.

### Model application in practice

In a simulated calculation where the BSA and CrCl of patients were assumed as 1.7 m^2^ and 89.5 ml/min, respectively, similar exposures of PEM (AUC_0–168h_: 106.31–106.38 mg h/L) were observed in patients with different allele mutations (C/C in ERCC1, T/C in CYP3A5, both, or none of them). Therefore, our results indicated that polymorphisms of CYP3A5 and ERCC1 only had significant effects on Q of PEM, and their impacts on PEM exposure were limited. On the contrary, PEM clearance and exposure were significantly affected by individual CrCl. We performed a simulation to predict the exposures of PEM under various dosing regimens in patients with different renal functions, in which the target exposure for PEM was defined as 164 mg h/L (de Rouw et al., 2019).

**FIGURE 5:**
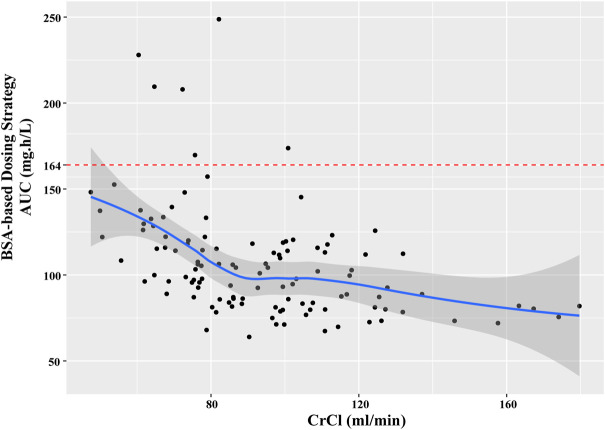
The relationship between estimated AUC and CrCl in current patients with BSA-based dosing strategy.

**FIGURE 6:**
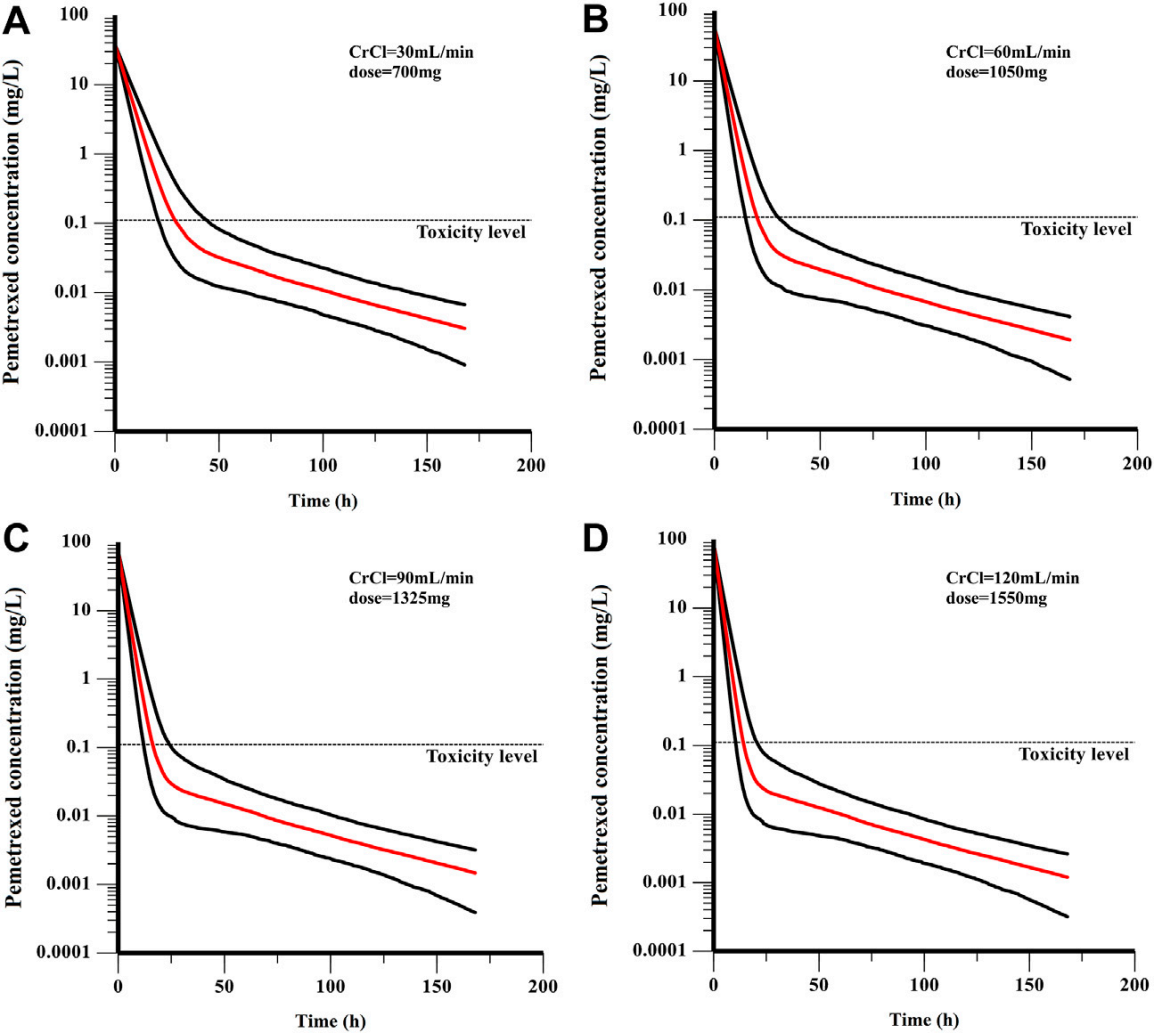
The PEM concentration-time curves of various dosing paradigms for patients with different renal function: **(A)** CrCl = 30 mL/min, dose = 700 mg; **(B)** CrCl = 60 mL/min, dose = 1050 mg; **(C)** CrCl = 90 mL/min, dose = 1325 mg; **(D)** CrCl = 120 mL/min, dose = 1550 mg. The red line is the median of the simulated concentration, and the black lines represent the 10th and 90th percentiles of the simulated data.

In the current study, all the patients were administrated with PEM with a BSA-based dosing (500 mg/m^2^ BSA) strategy, which is the prevailing method in clinical practice. However, our results found that majority of patients failed to achieve the target AUC, which was more obviously for those with higher CrCl (Figure 5). Specifically, the AUC of patients decreased with the elevation of CrCl, demonstrating that BSA-based dosing strategy was not suitable for patients with different renal function. As shown in Table 3, we proposed that dosing regimens need to be adjusted based on the varying CrCl levels to attain a similar median exposure with target AUC. The dosage of 700, 1050, 1325 and 1550 mg were recommended for patients with CrCl of 30, 60, 90 and 120 ml/min, respectively. Subsequently, the PEM concentration-time curves (corresponding to θ_ERCC1_ = 0 and θ_CYP3A5_ = 0) of various dosing paradigms mentioned above were conducted to inspect the time of PEM concentration below the toxicity threshold (0.110 mg/L) (Boosman et al., 2021). The results indicated that the PEM concentration would fall below the threshold within 24–36 h after administration (Figure 6), which meant above dosing paradigms might not bring additional toxicity risk for vitamin-supplemented patients as hypothesized.

**TABLE 3:** The simulated PEM exposure of various dosing regimens in patients with different renal function.

Group	Dosage	Simulated median of AUC	95% CI
CrCl = 30 ml/min	700	165.41	92.08–248.72
	850	200.86	111.81–302.02
	900	212.67	118.39–319.79
CrCl = 60 ml/min	700	110.54	61.54–166.22
	850	134.23	74.72–201.83
	1,050	165.81	92.30–249.32
CrCl = 90 ml/min	850	106.03	59.03–159.44
	1,200	149.70	83.33–225.09
	1,325	165.29	92.01–248.54
CrCl = 120 ml/min	850	89.70	49.93–134.88
	1,200	126.64	70.50–190.42
	1,550	163.57	91.06–245.96

## Discussion

In our study, 116 Chinese primary advanced NSCLC patients with a median age of 57 years (range 27–73 years) were included in the PPK analysis. The PEM concentrations of 192 plasma samples were detected by using UPLC-ESI-MS/MS and 116 blood samples of patients were genotyped for polymorphisms. The PPK model of PEM in Chinese primary advanced NSCLC patients was successfully built by applying NLME software.

Firstly, a two-compartment structural model with constant rate input (intravenous infusion) and first-order elimination was built to describe the concentration-time points of PEM. A proportional model and an exponential model with the smallest OFV were employed to illustrate the intra-individual and inter-individual variability of the PPK parameters of PEM, respectively. After establishing the basic model, a wide variety of covariates including 17 continuous covariates and 45 categorical covariates were searched for their effects on the PPK parameters of PEM. Our covariate analyses showed that CrCl was positively correlated with CL. V1 and V2 were not affected by any of searched covariates. A prospective cohort study on 106 advanced NSCLC patients treated with PEM in the Netherlands conducted by S. Visser reported a relation between CL of PEM and estimated glomerular filtration rate (eGFR) (Visser et al., 2019). It has been reported that 81% of PEM is bound to plasma proteins and only the unbound fraction could be filtered *via* glomerulus (de Rouw et al., 2021a). Active tubular secretion may remarkably contribute to the renal elimination of PEM. In our study the strong correlation between CrCl and CL is entirely in accordance with the elimination pathway of PEM. Both the PPK studies of PEM on NSCLC patients in the India (Srinivasan et al., 2019) and the United States (Latz et al., 2006) also indicated that CrCl was a significant covariate with respect to CL.

Although multiple PPK studies of PEM have been conducted in different countries, the effect of genetic variation on the pharmacokinetics of PEM is still unknown. Our study, for the first time, comprehensively evaluated the influence of genetic polymorphisms on pharmacokinetics behavior of PEM in Chinese NSCLC patients. We included genes of transporters (such as SLC19A1, SLC22A8, SLC22A9, SLC22A11, ABCB1 and ABCC2) and metabolic enzymes (such as CYP3A4, CYP3A5 and CYP19A1), as well as PEM-related genes (such as MTHFR, DHFR, TYMS, GGH, DCK, ATIC, and FOLR3). In addition, we also included the XRCC1, RRM1, ERCC1, ERCC2, and ERCC5 genes associated with platinum-based chemotherapy.

Importantly, it has been well established that SLC22A8 (also known as OAT3) is a renal transporter involved in the disposition of PEM (Srinivasan et al., 2019). In a recent study, researchers speculated that SLC22A8 polymorphism may cause inter-individual variability in PEM, but due to lack of genetic information, this part of the work has not been completed (Srinivasan et al., 2019). In our research, the CBT-PMRA gene chip provided information on 33 mutation sites of SLC22A8 (such as rs11231300, rs11231305, rs11568496, rs11568479, rs4149183, etc.). However, the covariate search of the stepwise procedure showed that none of them was significantly related to the inter-individual variability of PEM. Recently, the International Transporter Alliance published an article stating that due to the low genetic and functional diversity of the coding region, the mutation of the SLC22A8 transporter may not have a substantial impact on the individual differences in the elimination of xenobiotics by the kidney (Yee et al., 2018), which was consistent with our conclusion. In addition, other transporters including SLC22A9, SLC19A1, SLC22A11, ABCB1 and ABCC2 also did not affect the elimination of PEM.

Despite that transporters did not manifest expectant influence on the pharmacokinetics behavior of PEM, it was interesting to find that the rs776746 variant on CYP3A5 gene, which belongs to the hepatic metabolic enzyme subtypes, significantly affected the intercompartmental clearance of PEM, with the T/C genotype showing higher Q value than others, which was the first time that gene polymorphism was discovered to affect the PPK parameter of PEM. It has been demonstrated that rs776746 variant causes a deficiency of CYP3A5 enzyme activity due to a splice site which results in a truncated inactive enzyme (Alvarez-Elías et al., 2016). A previous study showed that there existed a significant association between rs776746 polymorphism of CYP3A5 and hypertension in Chinese Han population (Li et al., 2017). This SNP mutation has been suggested to be associated with decreased bioavailability of tacrolimus (Alvarez-Elías et al., 2016). In addition, the variant of rs776746 is also related to the prognosis of NSCLC patients (Jiang et al., 2016). However, no study has explored the effect of this variant on PEM behavior. To the best of our knowledge, for the first time, we find that CYP3A5 rs776746 polymorphism significantly affected the distribution speed of PEM after intravenous injection. The underlying cause is not yet clear, but it may be due to affecting the binding of PEM and plasma proteins in the body, or the penetration of PEM into the blood vessel wall.

Meanwhile, our covariate analyses showed that ERCC1 phenotype (rs3212986 polymorphisms) also significantly affected intercompartmental clearance of PEM. It has been suggested that interindividual genetic variability in drug metabolism, transport, target and DNA repair pathways could be applied as a blood biomarker to guide selection of therapy (Ingelman-Sundberg et al., 2018). ERCC1 is a gene encoding for proteins of the nucleotide excision repair complex, which is a group of proteins to repair DNA (Kim et al., 2020). With regard to ERCC1 SNPs in NSCLC patients, two SNPs have been commonly investigated, namely ERCC1 C8092A SNP (rs3212986) and ERCC1 T19007C SNP (rs11615) (Grenda et al., 2020). SNP (rs3212986) seems to affect the stability of ERCC1 mRNA, while the SNP (rs11615) may influence the levels of ERCC1 mRNA. In general, polymorphisms in DNA repair genes may result in interindividual variability of the capacity to repair DNA (Liao et al., 2018). When compared with the variant genotypes (C/A + A/A), the wild-type ERCC1 (C/C) in rs3212986 locus was reported to exhibit longer median progression free survival among NSCLC patients with platinum-based chemotherapy (Grenda et al., 2020). However, in the current study, we found that ERCC1 polymorphisms could also affect the *in vivo* pharmacokinetic manner of PEM distribution, with the wild-type ERCC1 (C/C) in rs3212986 locus exhibiting higher Q values than the other two variant genotypes. We speculated that the gene ERCC1 might have an effect on PEM disposition between the two compartments by influencing the *in vivo* binding process of PEM with plasma protein (Papachristos et al., 2020). In addition, a recent article demonstrated that ERCC1 mutation could hinder the repair process of DNA damage and lead to dysfunction of liver and kidney (Apelt et al., 2021). We speculated that the mutation of rs3212986 might alter the function of liver-mediated metabolism or kidney-mediated transport, resulting in altered Q values in patients with different variants of ERCC1. Our study demonstrated that PPK analyses that add genetic data as covariates, appear to a potential approach to study the efficacy and safety profile of drug.

Taken together, our study suggested that a two-compartment model with first-order elimination was the best fit for PPK of PEM in Chinese primary advanced NSCLC patients, along with CrCl as an essential covariate on CL, ERCC1 (rs3212986) and CYP3A5 (rs776746) gene polymorphisms as essential covariates on Q. The prediction accuracy of the final model was reliable and the stability of the final model was good, which were indicated *via* GOF plots, non-parametric bootstrap and VPC. The population typical values of V1, V2, CL and Q were 18.94 L, 5.12 L, 8.29 L/h, and 0.10 L/h, respectively. PPK models of PEM in NSCLC patients in the Netherlands (Visser et al., 2019), India (Srinivasan et al., 2019) and the United States (Latz et al., 2006) have also been reported. In detail, the typical PPK values of V1, V2, CL and Q were 15.9 L, 21.6 L, 4.58 L/h and 0.05 L/h for Netherlandish population, 5.2 L, 5.9 L, 3.3 L/h and 6.8 L/h for Indian population, as well as 12.9 L, 3.38 L, 5.5 L/h and 0.86 L/h for American population, respectively. The typical PPK values of PEM varied significantly in different studies, probably due to the ethnic differences in the study population.

At last, we simulated and predicted the exposure of PEM under different dosing strategies, and then compared it with the reported data to infer effective or toxic results. Comparable with most chemotherapeutic drugs, BSA-based dosing strategy as the standard-of-care was conventionally applied to adjust the dosage of PEM (Faber et al., 2022). But, the simulation analysis of our study showed that the AUC of patients decreased with the elevation of CrCl when BSA-based dosing strategy was applied. In detail, it meant that patients with attenuated renal function would have a higher PEM exposure of AUC and a greater probability of result in intolerable adverse reactions, such as neutropenia and thrombocytopenia, than those with normal renal function. Renal impairment is a greatly prevalent symptom among NSCLC patients (de Rouw et al., 2019; Hill et al., 2019). During the maintenance therapy of PEM, advanced NSCLC patients are at risk to result in renal impairment (Malyszko et al., 2017; Visser et al., 2018). Although PEM is primarily excreted *via* kidney, renal function is not taken into account by BSA-based dosing regimen (de Rouw et al., 2019; de Rouw et al., 2021a). Of note, our PPK study of PEM suggested that renal function (CrCl), as the main covariate of CL, might be a more reliable reference factor than BSA to guide the dose regimen. The simulation results showed that a lower dosage was recommended for patients with impaired renal function to attain a target exposure. Renal function-based dosing adjustment exhibited a stable PEM exposure, which is consistent with previous reports (Visser et al., 2019).

In the present study, a few given dosing regimens in Table 3 were higher than the normal BSA-based dosing, especially for patients with elevated renal function. It was considered to associate with the higher typical value of CL in current population than previous reports (Latz et al., 2006). Considering the potential toxicity risk, the PEM concentration-time curves of recommended dosing paradigms were simulated based on the lowest Q (θ_ERCC1_ = 0 and θ_CYP3A5_ = 0). It has been reported that PEM toxicity is related to the time above threshold concentration which is 0.110 mg/L for vitamin-supplemented patients (Boosman et al., 2021). It was suggested to monitor the PEM concentration from 24 to 36 h after administration for toxicity management (de Rouw et al., 2021b). Through a simulation of concentration-time curve, we speculated that dosing regimens recommended for patients with different CrCl levels, might not bring additional toxicity risk for these patients if they receive vitamin-supplement. But it still needed to be confirmed by further clinical evidence.

In our current study, there were several limitations. Firstly, due to the limited sample size, the final PPK model of PEM in Chinese primary advanced NSCLC patients was not externally confirmed. An independent group should be added in the future study. Besides, the benefit of the optimized pharmacokinetically-guided PEM dosing strategy was only simulated based on PPK analyses, which needs to be further validated in a randomized clinical trial. Moreover, future trials might expand to the combined administration of PEM and advanced therapeutics, such as immunotherapy and targeted therapy.

## Conclusion

In summary, this is the first study to characterize the PPK of PEM in Chinese primary advanced NSCLC patients. A two-compartment model with first-order elimination was built, along with CrCl as a significant covariate on CL, ERCC1 (rs3212986) and CYP3A5 (rs776746) gene polymorphisms as essential covariates on Q. Most importantly, our study proposed that dose adjustment of PEM based on renal function, rather than BSA, might be an optimal strategy for this particular population.

## Data Availability

The data presented in the study are deposited in the NCBI repository, BioProject: PRJNA864251, https://dataview.ncbi.nlm.nih.gov/object/PRJNA864251. The SNP data can be found in the Supplementary Material.
