# Supplementary material for: Population pharmacokinetic study of pemetrexed in chinese primary advanced non-small cell lung carcinoma patients
Source: Front Pharmacol. 2022 Aug 25;13:954242. doi: 10.3389/fphar.2022.954242 (PMC9466465; doi:10.3389/fphar.2022.954242)
Supplement: Supplementary file 1 [file Table2.docx]

Supplementary Table 2

Supplemental Table 2. Gene polymorphism SNP locus in stepwise process

| SNP gene locus | Distribution of different genotypes |
| --- | --- |
| rs10897315 (SLC22A8) | (A/A):(A/G):(G/G)=24:56:36  57.0 (27.0-73.0) |
| rs11231300 (SLC22A8) | (G/G):(T/G)=109:7  7 |
| rs11231305 (SLC22A8) | (A/A):(C/C):(A/C)=11:60:45  37.7 (27.4-50.9) |
| rs4149183 (SLC22A8) | (A/A):(A/G):(G/G)=51:50:15 |
| rs948982 (SLC22A8) | (G/G):(T/T):(T/G)=78:1:37 |
| rs11231299 (SLC22A8) | (A/A):(A/G):(G/G)=12:52:52 |
| rs10750978 (SLC22A8) | (G/G):(C/C):(C/G)=71:5:40 |
| rs7926184 (SLC22A8) | (G/G):(T/T):(T/G)=77:7:32 |
| rs58077467 (SLC22A8) | (A/A):(A/G):(G/G)=79:34:3 |
| rs74189618 (SLC22A8) | (A/A):(A/G):(G/G)=84:31:1 |
| rs10792367 (SLC22A8) | (G/G):(C/C):(C/G)=34:32:50 |
| rs11568482 (SLC22A8) | (T/T):(A/T)=103:13 |
| rs45438191 (SLC22A8) | (A/A):(A/G)=115:1 |
| rs7113486 (SLC22A8) | (A/G):(G/G)=6:110 |
| rs4149181 (SLC22A8) | (A/A):(A/G):(G/G)=104:11:1 |
| rs4578390 (SLC22A8) | (A/G):(G/G)=26:90 |
| rs1801133 (MTHFR) | (A/A):(A/G):(G/G)=18:57:41 |
| rs1650697 (DHFR) | (A/A):(A/G):(G/G)=23:47:46 |
| rs699517 (TYMS) | (C/C):(T/C):(T/T)=12:58:46 |
| rs11545078 (GGH) | (A/A):(A/G):(G/G)=1:21:94 |
| rs10464903 (GGH) | (C/C):(T/C):(T/T)=45:53:18 |
| rs2228001 (XPC) | (G/G):(T/G):(T/T)=17:52:47 |
| rs12995526 (ATIC) | (C/C):(T/C):(T/T)=76:34:6 |
| rs1799782 (XRCC1) | (A/A):(A/G):(G/G)=13:41:62 |
| rs2306283 (SLCO1B1) | (A/A):(A/G):(G/G)=3:40:73 |
| rs4149056 (SLCO1B1) | (C/C):(T/C):(T/T)=2:31:83 |
| rs4149015 (SLCO1B1) | (A/A):(A/G):(G/G)=3:30:83 |
| rs4149117 (SLCO1B3) | (G/G):(T/G):(T/T)=64:45:7 |
| rs11045585 (SLCO1B3) | (A/A):(A/G):(G/G)=79:31:6 |
| rs7311358 (SLCO1B3) | (A/A):(A/G):(G/G)=64:45:7 |
| rs232043 (RRM1) | (A/A):(A/G):(G/G)=66:41:9 |
| rs1128503 (ABCB1) | (A/A):(A/G):(G/G)=46:50:20 |
| rs3740066 (ABCC2) | (C/C):(T/C):(T/T)=75:35:6 |
| rs2273697 (ABCC2) | (A/A):(A/G):(G/G)=1:20:95 |
| rs12762549 (ABCC2) | (C/C):(C/G):(G/G)=25:54:37 |
| rs717620 (ABCC2) | (C/C):(T/C):(T/T)=75:35:6 |
| rs11615 (ERCC1) | (A/A):(A/G):(G/G)=9:47:60 |
| rs3212986 (ERCC1) | (A/A):(A/C):(C/C)=15:56:45 |
| rs1799793 (ERCC2) | (C/C):(T/C):(T/T)=110:5:1 |
| rs1047768 (ERCC5) | (C/C):(T/C):(T/T)=9:51:56 |
| rs28371759 (CYP3A5) | (A/A):(A/G)=114:2 |
| rs776746 (CYP3A5) | (C/C):(T/C):(T/T)=60:49:7 |
| rs4646 (CYP19A1) | (A/A):(A/C):(C/C)=11:47:58 |
| rs1695 (GSTP1) | (A/A):(A/G):(G/G)=76:35:5 |
