# Supplementary material for: Population pharmacokinetic study of pemetrexed in chinese primary advanced non-small cell lung carcinoma patients
Source: Front Pharmacol. 2022 Aug 25;13:954242. doi: 10.3389/fphar.2022.954242 (PMC9466465; doi:10.3389/fphar.2022.954242)

Supplementary Information

# Inclusion/exclusion criteria

1.1 Inclusion criteria:

(1) Newly diagnosed primary non-small cell lung cancer；

(2) Adopt pemetrexed or pemetrexed/platinum chemotherapy；

(3) Men over 18 years old and non-pregnant, non-lactating women；

(4) The expected survival time is greater than or equal to 12 weeks, and the physical status ECOG rating is 0-2.

1.2 Exclusion criteria:

Combining radiotherapy or using targeted agents/immunotherapy before and during chemotherapy affects the evaluation of the efficacy of pemetrexed.

# Analytical method validation

2.1 Specificity

Six blank plasmas from different sources were taken to investigate the specificity of pemetrexed. The experimental results confirmed that the plasma matrix and other endogenous substances, internal standards and tested compounds would not interfere with each other (SI Figure 1), which proved that the detection method can be used to detect pemetrexed.


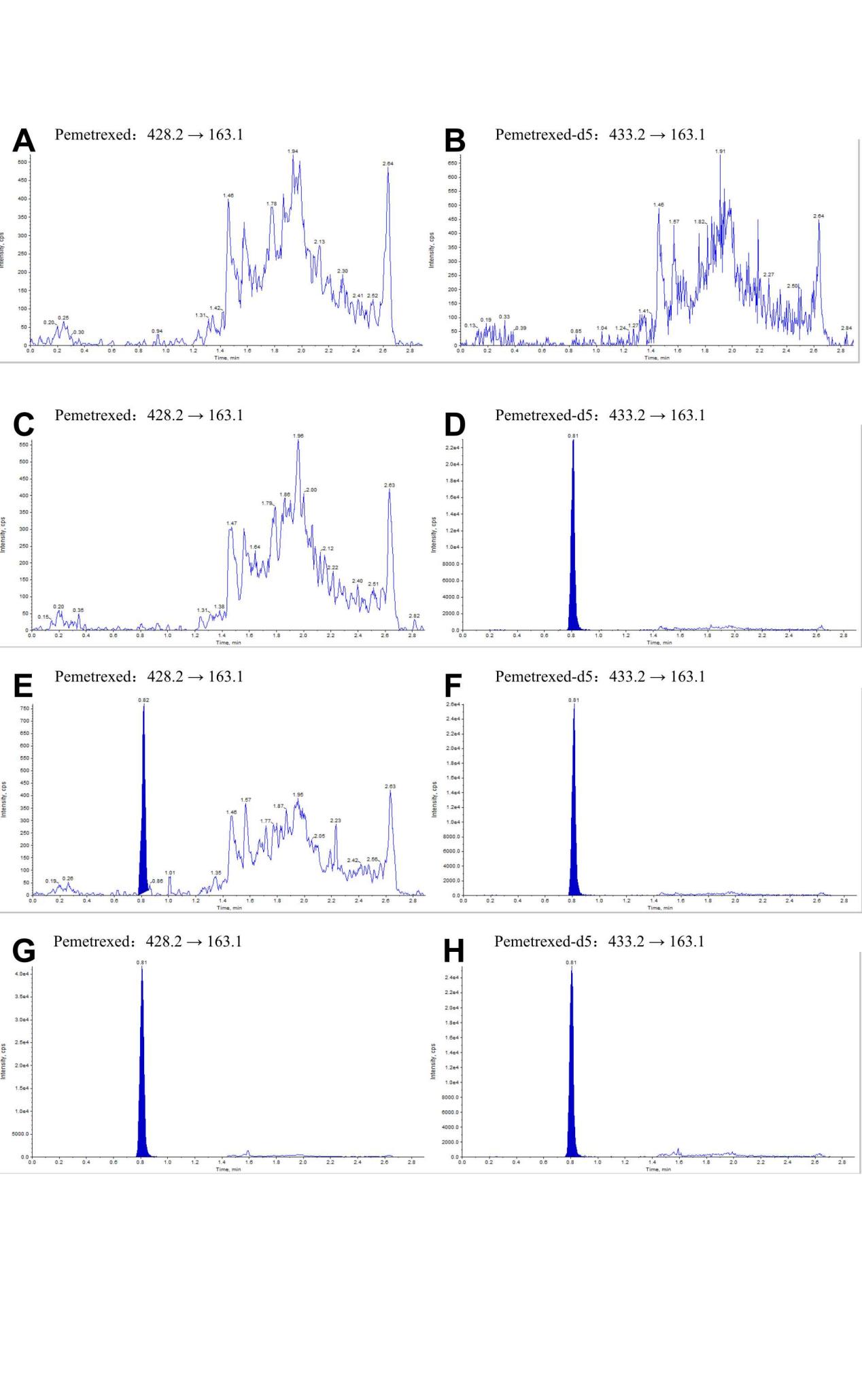


SI Fig 1. LC-MS chromatograms of different samples. (A&B) Blank plasma sample; (C&D) Blank plasma sample with internal standard; (E&F) Lower limit of quantitation sample; (G&H) Upper limit of quantification sample.

2.2 Lower limit of quantification and standard curve

The standard curve samples of pemetrexed were prepared, and the drug concentrations were 600, 540, 300, 240, 60.0, 15.0, 5.00, and 2.50 ng⋅mL^-1^. After sample injection analysis, take the pemetrexed plasma concentration (X) as the abscissa, the ratio of the peak area of pemetrexed to the peak area of the internal standard (Y) as the ordinate, and use 1/x^2^ as the weighting coefficient to perform linear regression to obtain a regression equation: Y=0.00257*X+0.00152, R^2^=0.9980, the linear relationship is good, and the lower limit of quantification is 2.50 ng⋅mL^-1^.


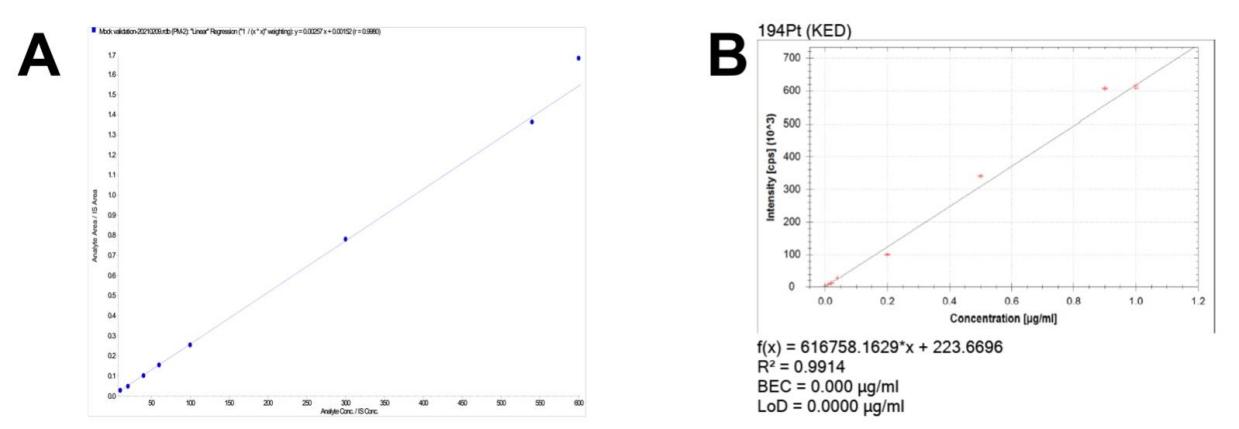


SI Fig 2. Standard curve of pemetrexed.

2.3 Accuracy and precision

Prepare pemetrexed samples of lower limit of quantification (LLOQ), low concentration quality control (LQC), medium concentration quality control (MQC), and high concentration quality control (HQC), followed by injection and analysis. Each concentration is prepared in parallel (n=6). Calculate the intra-batch accuracy and precision, and then continuously measure 3 batches of samples to calculate the inter-batch precision. As shown in SI Table 1, the accuracy of pemetrexed is between 85% and 115%, and the intra-assay and inter-assay precisions are both less than 15%, which are in line with the detection requirements for biological samples.

SI Table 1. Accuracy and precision results

|  |  | Intra-batch（n=6） | | | Inter-batch（n=3） |
| --- | --- | --- | --- | --- | --- |
|  | Nominal concentration | Measure concentration | Accuracy% | Precision% | Precision% |
| Pemetrexed（ng⋅mL^-1^） | 10.0 (LLOQ) | 10.0 ± 1.1 | 0.4 | 11.0 | 10.0 |
|  | 30.0 (LQC) | 31.0 ± 1.6 | 3.4 | 5.1 | 6.7 |
|  | 180 (MQC) | 167 ± 3.6 | -7.1 | 2.2 | 7.7 |
|  | 450 (HQC) | 444 ± 19.8 | -1.2 | 4.4 | 7.1 |

2.4 Recovery rate and matrix effect

Prepare pemetrexed samples of LQC, MQC and HQC, and analyze the peak area as A. In addition, take blank plasma without adding working solution, get the blank matrix extract, then add the LQC, MQC and HQC working solutions of pemetrexed, and analyze the sample, the peak area is marked as B. The peak area of LQC, MQC and HQC working solution injection analysis is denoted as C.

The extraction recovery rate is calculated by A/B. The extraction recovery rate of pemetrexed LQC, MQC and HQC is 95.2%~123.2%, and the RSD is less than 15%. The matrix effect is calculated by B/C, the matrix effect of pemetrexed LQC, MQC and HQC is 87.5%-95.6%, RSD is less than 15%.

SI Table 2. Results of extraction recovery and matrix effect

|  |  | extraction recovery （n=6） | | matrix effect（n=6） | |
| --- | --- | --- | --- | --- | --- |
|  |  | Mean/% | RSD/% | Mean/% | RSD/% |
| Pemetrexed（ng⋅mL^-1^） | 30.0 (LQC) | 103.4 | 5.7 | 87.5 | 4.3 |
|  | 180 (MQC) | 123.2 | 3.7 | 95.6 | 5.1 |
|  | 450 (HQC) | 95.2 | 5.1 | 93.8 | 4.8 |

2.5 Stability

Pemetrexed stability of dilution, repeated freezing/thawing for 3 times, 12-h room temperature, and at -80°C for 1 month were evaluated. Consistent with previous studies [1-3], the relative errors of all sample concentrations are within ±15%, which proves that the samples are stable.

References:

1. Bobin-Dubigeon, C., et al., *Development and validation of an improved liquid chromatography-mass spectrometry method for the determination of pemetrexed in human plasma.* J Chromatogr B Analyt Technol Biomed Life Sci, 2009. **877**(24): p. 2451-6.

2. Rivory, L.P., et al., *Highly sensitive analysis of the antifolate pemetrexed sodium, a new cancer agent, in human plasma and urine by high-performance liquid chromatography.* J Chromatogr B Biomed Sci Appl, 2001. **765**(2): p. 135-40.

3. Stoop, M.P., et al., *A new quantification method for assessing plasma concentrations of pemetrexed and its polyglutamate metabolites.* J Pharm Biomed Anal, 2016. **128**: p. 1-8.

# Covariate screen and final model development process

SI Table 3. Covariate screen and final model development process^*^

| Step | Covariate screened | OFV | △OFV | P value | Comments |
| --- | --- | --- | --- | --- | --- |
| 1 | None | 338.401 |  |  | Base model |
| 2 | Cl-CrCl | 310.574 | 27.827 | <0.001 | Covariate model |
| 3 | Cl-CrCl, Q-rs3212986 | 298.398 | 12.176 | <0.001 | Covariate model |
| 4 | Cl-CrCl, Q-rs3212986, Q-rs776746 | 294.246 | 4.152 | <0.05 | Full model |
| 5 | Cl-CrCl, Q-rs3212986, Q-rs776746, remove V1/V2 random effects | 289.683 | 4.563 | <0.05 | Final model |

*OFV, objective function value; ΔOFV, change of objective function value; CL, clearance; Q, intercompartment clearance; V, apparent volume of distribution; CrCl, creatinine clearance.

# Outlier handling

In developing the PPK base model, we found that there existed two outliners in total 194 dots in the figure of concentration vs PRED. In order to improve the accuracy and efficacy of PPK model, we deleted the two abnormal values in the base model, and the final model showed satisfactory results.


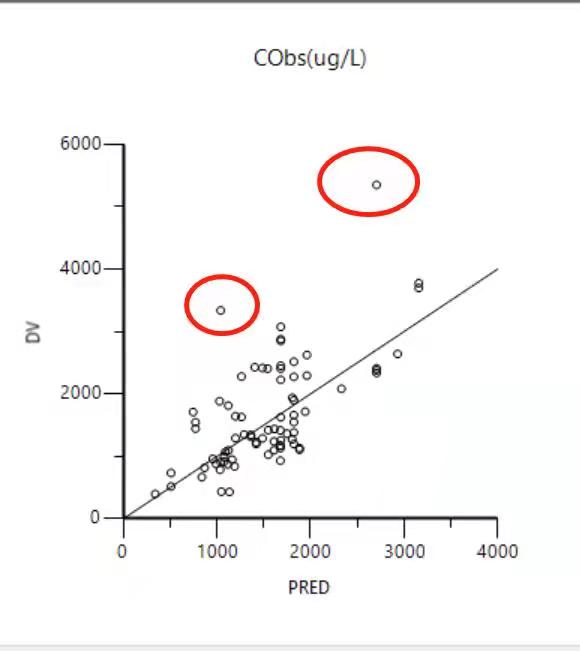

Supplement: Supplementary file 3 [file DataSheet1.docx]
